# Ant colony optimization-based firewall anomaly mitigation engine

**DOI:** 10.1186/s40064-016-2489-6

**Published:** 2016-07-08

**Authors:** Ravi Kiran Varma Penmatsa, Valli Kumari Vatsavayi, Srinivas Kumar Samayamantula

**Affiliations:** MVGR College of Engineering, Vizianagaram, AP India; Andhra University College of Engineering, AU, Visakhapatnam, AP India; University College of Engineering, JNTU Kakinada, Kakinada, AP India

**Keywords:** Packet-filter firewall rules, Ant colony optimization (ACO), Metaheuristics, Shadowing, Generalization, Correlation, Redundancy, Rule anomalies, Rule reordering

## Abstract

A firewall is the most essential component of network perimeter security. Due to human error and the involvement of multiple administrators in configuring firewall rules, there exist common anomalies in firewall rulesets such as Shadowing, Generalization, Correlation, and Redundancy. There is a need for research on efficient ways of resolving such anomalies. The challenge is also to see that the reordered or resolved ruleset conforms to the organization’s framed security policy. This study proposes an ant colony optimization (ACO)-based anomaly resolution and reordering of firewall rules called ACO-based firewall anomaly mitigation engine. Modified strategies are also introduced to automatically detect these anomalies and to minimize manual intervention of the administrator. Furthermore, an adaptive reordering strategy is proposed to aid faster reordering when a new rule is appended. The proposed approach was tested with different firewall policy sets. The results were found to be promising in terms of the number of conflicts resolved, with minimal availability loss and marginal security risk. This work demonstrated the application of a metaheuristic search technique, ACO, in improving the performance of a packet-filter firewall with respect to mitigating anomalies in the rules, and at the same time demonstrated conformance to the security policy.

## Background

**Fig. 1:**
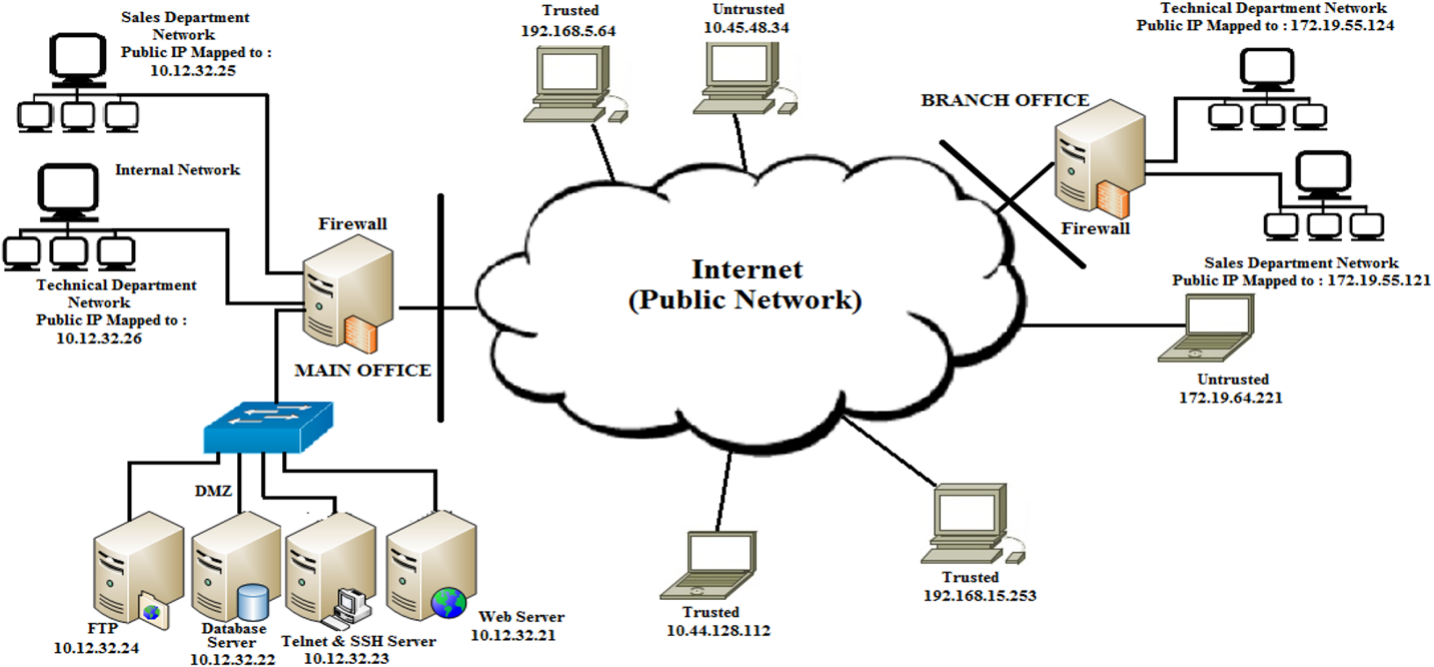
Typical organizational firewall placement scenario (Policy 1)

A firewall is one of the most vital network defense components that can be used to filter unsolicited traffic. A packet-filter firewall can filter packets based on fields in the network layer and transport layer such as the source internet protocol (IP) address, destination IP address, source and destination port address, and protocol field. The firewall will perform its job of filtering packets based on a set of rules written by the administrator. The number of rules and the traffic that must be filtered depend upon the organization’s security policy. A firewall is typically placed at the network perimeter as shown in Fig. 1. A typical scenario consists of an internal network, a de-militarized zone (DMZ), the Public Network, and a Branch Office network. The internal network must be protected from the public network. The DMZ contains systems and servers that normally are meant for public access. There can be trusted and non-trusted or blacklisted hosts in the public network.

The security policy that must be enforced at the main office firewall is shown in Table 1. The firewall ruleset that was evolved by the administrator in the process of implementing the security policy at main office firewall is listed in Table 2.

**Table 1:** Security policy to be implemented at main office firewall of Fig. 1

1. Full access is to be given to the organization telnet server for the organization administrator’s home IP “10.44.128.112”
2. Full access is to be given to the organization telnet server for the organization’s work from home IP address “192.168.5.64”
3. Full access is to be given to the organization telnet server for the organization’s work from home IP address “192.168.15.253”
4. The technical department of the branch office having IP “172.19.55.124” must have access to the entire organization’s internal network
5. The Sales Department of the branch office having IP “172.19.55.121” must have access to web server “10.12.32.21” and ftp server “10.12.32.24” of the organization only
6. Access to webserver http port “10.12.32.21: 80” must be open to all
7. All remaining access from untrusted source “172.19.55.122” except http connection to web server is to be stopped
8. All remaining access from untrusted source “172.19.55.123” except http connection to web server is to be stopped
9. All remaining access from untrusted source “10.45.48.34” except http connection to web server is to be stopped
10. All remaining access from untrusted source “172.19.64.221” except http connection to web server is to be stopped

**Table 2:** Initial configuration of firewall rules (Policy 1)

Rule	Protocol	Source IP	Source port	Destination IP	Destination port	Action
1	*	172.19.55.124	*	10.12.32.21–10.12.32.22	1–80	Allow
2	TCP	172.19.55.*	*	10.12.32.21	80	Allow
3	TCP	192.168.5.64	*	10.12.32.23	23	Allow
4	*	172.19.55.121–172.19.55.124	*	10.12.32.*	*	Allow
5	*	10.45.48.34	*	10.12.32.*	*	Deny
6	*	10.*.*.*	*	10.12.32.21	80	Allow
7	TCP	172.19.55.121	*	10.12.32.24	20–21	Allow
8	TCP	172.19.55.121	*	10.12.32.21	80	Allow
9	*	172.19.55.121	*	10.12.32.*	*	Deny
10	TCP	192.168.15.253	*	10.12.32.23	23	Allow
11	TCP	10.44.128.112	*	10.12.32.23	23	Allow
12	*	172.19.55.122	*	10.12.32.*	*	Deny
13	*	172.19.55.123	*	10.12.32.*	*	Deny
14	*	172.19.64.221	*	10.12.32.*	*	Deny

### Firewall anomalies

Often, more than one administrator might be involved in writing or appending rules to the firewall. When the size of the ruleset grows, the complexity and interdependency of the rules increase and might be susceptible to errors, which can lead to anomalies. These anomalies are often unavoidable due to human error and often cannot be detected and corrected manually. There are four types of anomalies in packet-filter firewalls (Al-Shaer and Hamed 2004a, b; Yuan et al. 2006), as follows: (1) shadowing, (2) generalization, (3) correlation and (4) redundancy. Among the four mentioned anomalies, shadowing, generalization and correlation considered “policy conflicts” because these anomalies are caused by rules with interdependency but that have different actions. However, Redundancy exists because of rules with interdependencies that have the same action.

#### Shadowing

A rule is considered shadowed by one or a set of preceding rules if each packet matched by this rule is also matched by those preceding rules but takes a different action. For example in the ruleset showed in Table 2, rules “9, 12, and 13” are shadowed by rule “4.” That is, any packet that matches any one of rules “9, 12, and 13” whose actions are “Deny” also matches rule “4,” whose action is “Allow.”

#### Generalization

A rule is considered a generalized rule if it is a superset of any preceding rules but with a different action. For example, rule “9” is a generalized rule of rules “7 and 8.” Rule 9’s action is Deny whereas Rule 7 and 8’s actions are Allow.

#### Correlation

Two rules are considered correlated if they have an intersection of rule spaces, i.e., one rule is a superset to the other in some part of the fields and vice versa. Rule pairs (6, 5), (9, 2), (12, 2), and (13, 2) are correlated from Table 2.

#### Redundancy

A rule is declared redundant if the action taken by the firewall on a packet does not change even when it is removed from the ruleset. For example, rule “8” in Table 2 is redundant because the set of packets matched to rule “8” also matches to rule “2” and both perform the same action, i.e., “Allow.” Therefore, removing or moving rule “8” will not affect the firewall.

These anomalies often results in unintended behavior and may cause loss of availability and/or security risk to the organizational resources. Therefore detection and resolution of such anomalies in an efficient way is very much essential for any organization.

The remainder of this article is arranged as follows: “Related work” section presents the previous work in the domain along with their limitations and also the highlights of our proposed solution. “Anomaly detection methodology” section discusses anomaly detection technique using segmentation and grid representation process with an example. The role of “Action Constraint Generation” (ACG) and Strategies used for ACG in the process of resolving anomalies are discussed in “Anomaly resolving” section. The proposed modifications, primarily the new Trust Factor (TF)-based ACG, the role of ACO in anomaly resolving and reordering, and the adaptive reordering algorithm are provided in “ACOFAME” section. The experimentation results, analysis and comparison with the existing system are presented in “Experimentation results and comparison” section, and conclusion and future scope are provided in the last section.

## Related work

An earlier study by Wool (2004) revealed that corporate firewalls suffer from poor configurations. The author analyzed rulesets gathered from several corporate firewalls and found that misconfigurations in framing rules caused breaches of security policies. Wool (2010) also proved that the number of errors is correlated to the ruleset complexity. Later, the same author analyzed several corporate firewalls and found that the same situation prevailed and misconfigurations in the firewall rules were ubiquitous.

The framework called “fast detect” (Hari et al. 2000) was one of the earlier works focused on detecting and resolving correlation conflicts by reordering rules. The authors addressed individual local conflicts; however, global conflicts were not addressed. They identified a circular looping problem among the conflicting rules, even after reordering. The authors proposed resolving filters to break circular loops within the rules to solve the problem. The fast-detect algorithm is not suitable for five-tuple firewalls because it is only based on two-tuples, source and destination IP addresses. The authors tried to address the five-tuple issue by maintaining three-tuples as constants. However, the solution was not feasible for real-valued five-tuples.

The authors of Al-Shaer and Hamed (2004a, b) made an effort to find the errors in the firewall rules of different organizations. They found that many such anomalies are unintended and mostly caused by human mistakes. Firewall anomalies are classified into four types: shadowing, generalization, correlation, and redundancy. The authors proposed anomaly detection with the help of a tree representation of the rules. A path from the root to the leaf node of one rule not colliding with that of another rule implies no anomaly. If the paths of two rules collide, then there exists an anomaly. The authors left the resolving part to the administrator for manual intervention. They also proposed a Firewall Policy Advisor that helps in editing the rule and guides the administrator in inserting a new rule at an appropriate position to remove anomalies. However, the results were not checked against security policy conformation.

The authors of Yuan et al. (2006), Benelbahri and Bouhoula (2007) and Al-Shaer and Hamed (2004a, b) have suggested methodologies to detect pairwise anomalies. The authors left the resolving part to the administrators, giving the details of the conflicts present in the rules. The authors of Yuan et al. (2006) designed a tool called “FIREMAN,” which tries to detect anomalies existing in stateless firewalls. They also tried to minimize the size of the policy by summarizing. The authors converted a firewall policy into a rule graph and used Binary Decision Diagrams (BDDs) to detect anomalies. The limitation is that the graph was able to detect only pairwise anomalies, in which it compares the present rule with preceding rules but not with succeeding rules.

In Benelbahri and Bouhoula (2007) the authors proposed an algebraic mathematical method for detecting anomalies. They also proposed a new language for writing rules by designing a compiler to parse the rules; however, the resolution was not addressed in that study. The authors in Muhammad et al. (2006) proposed pair-wise anomaly detection. Here, one rule is compared with another rule and the process takes action based on the relationship of the two rules. A semi-automated detection and resolving strategy of firewall anomalies was proposed that followed a default-deny policy for the packets that fell into the conflict space. The default-deny policy might increase the availability loss, although the policy might reduce the security risk. The author in Alex (2009) designed a tool to guide the user in entering new rules into existing firewalls without creating conflicts, but detection or resolving already-existing anomalies was not addressed.

The authors in Pozo et al. (2008) were able to detect global anomalies by dividing the rules into groups so that the administrator could easily address the smaller groups independently. The ruleset was initially converted to a potential conflict graph (PCG). Then, the PCG was divided into independent clusters of inconsistence rules (ICIR). However, the resolver was manual.

The authors in Liang et al. (2014) developed a firewall anomaly detector to identify shadowing and correlation using ordered binary decision trees. The authors provided a formal model to detect global conflicts in firewall policies. The model was able to detect both local and global conflicts efficiently. The ordered binary tree was able to reduce false positives. This study did not present any anomaly resolver.

“Firewall Anomaly Management Environment (FAME)” (Hu et al. 2012). was able to detect and resolve the anomalies in firewall policies both locally and globally. The authors used a “risk value-based combination algorithm” to reorder the rules semi-automatically to eliminate Shadowing, Generalization, and Correlation anomalies. FAME also had a redundancy viewer to eliminate redundancies in firewall policies; however, the viewer was addressed in a separate phase. In Hu et al. (2012), the rule space was initially divided into disjoint segments, and then a mapping was conducted to discover conflicted and non-conflicted segments. Once this mapping was performed, semi-automatic methods were used to reorder the rules to resolve the conflicts. The authors employed a “Common Vulnerability Scoring System (CVSS)-based Risk Level” to decide whether to Allow or Deny packets that belonged to a conflicted segment. The only means of resolving the conflicts or anomalies accidentally created by administrators was to follow techniques to reorder the rules. Permutation and Greedy algorithms were used in Hu et al. (2012) to select the best order of rules, which can avoid conflicts. However, although the permutation method produces the best order of rules, it is much too time consuming. Conversely, the greedy method was quick but might not produce the best-ordered result.

In the literature, additional works that have addressed firewall rule anomaly detection include Abbes et al. (2008), Ben Neji and Bouhoula (2011), Bouhoula et al. (2008), Matsumoto and Bouhoula (2008), Saadaoui et al. (2014). In Abbes et al. (2008) the authors proposed rule anomaly detection based on a tree structure that represented the rules. An inference system was used to construct the tree and to identify the anomalies. The tree structure helped to find the anomalies more quickly. However, no resolving techniques were proposed.

In Bouhoula et al. (2008), the authors proposed an efficient means of detecting firewall anomalies using a matrix-based method and also proposed methods to rectify those anomalies. In Matsumoto and Bouhoula (2008), the authors depicted the importance of security policy, and they proposed a method to verify the firewall rules against the policy. The work proposed in Saadaoui et al. (2014) is similar recent work that successfully addressed detection of all of the types of anomalies by dividing them into two categories: superfluous and conflicting. The authors used a two-dimensional grid in the detection phase, which was a simple and efficient detection technique. The authors of Saadaoui et al. (2014) also proposed a resolving method that would remove the shadowed rules; in the case of correlated rules, they inserted a new rule. However, this approach had limitations; occasionally, removing rules could violate the security policy, whereas adding rules increased the ruleset size.

In summary, several issues must be addressed in the case of packer-filter firewall anomaly mitigation. There is a need to investigate automated anomaly resolution techniques that can help to minimize human error. Another important issue is security policy conformation even after resolution of existing anomalies. There is also a need to research optimizing ruleset size by eliminating redundant rules.

### Contributions of this study

One solution to avoiding induced anomalies of the firewall rules and removing any redundant rules is reordering. Finding the best order of rules among all possible orders is a combinatorial optimization problem. To date, no one has studied the application of Ant Colony Optimization as a metaheuristic search to reorder packet-filter firewall rules.

This study proposes a framework with the following features:

1. Introduces a concept called “TF” to establish a relationship between the security policy and the anomaly resolver. TF helps in conforming to the security policy even after reordering.
2. Proposes methods to automate the anomaly mitigation process with the help of modified “Action Constraint Generation” strategies.
3. Applies ACO to generate an optimized set of ordered rules, which not only removes anomalies but also eliminates redundancy in this process.
4. Introduces an algorithm that adaptively handles a newly entered rule to avoid running the tool for the entire ruleset and hence save much time.

The proposed system will be called the ACO-based Firewall Anomaly Mitigation Engine (ACOFAME). The results of experimentation conducted on several rulesets have proved that our approach has improved firewall performance when measured with important evaluation parameters such as the number of conflicts resolved, availability loss and security risk.

## Anomaly detection methodology

A packet-filter firewall works on five fields. Each field has its range of values and can be considered operating in a five-dimensional continuous packet space. Manual identification of anomalies is very difficult. Several researchers have proposed methodologies to identify anomalies (Hu et al. 2012; Liang et al. 2014; Hari et al. 2000; Alex 2009; Pozo et al. 2008; Yuan et al. 2006; Benelbahri and Bouhoula 2007; Al-Shaer and Hamed 2004a, b). A BDD-based segmentation approach was used in Hu et al. (2012); the firewall-rule packet space was represented in the form of “Segmentation and Grid.”

### Segmentation

**Fig. 2:**
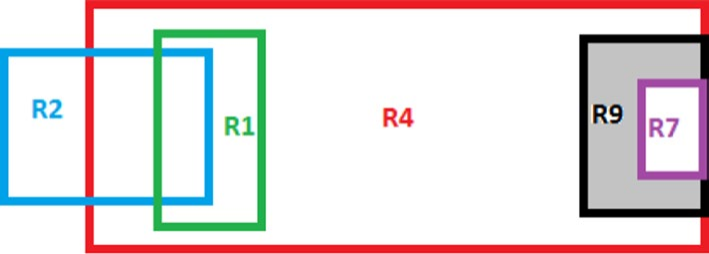
Example rule space

**Fig. 3:**
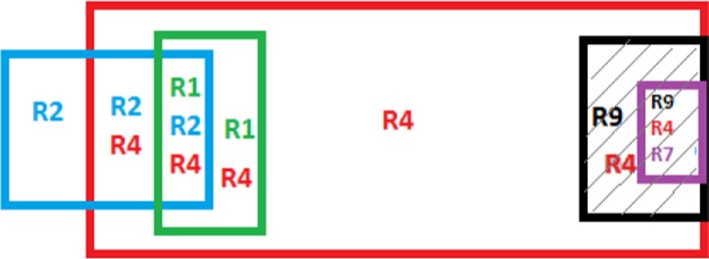
Overlapped rule space

**Fig. 4:**
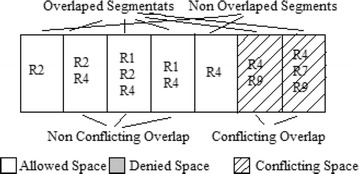
Formation of Segments from the rule space

Figures 2, 3 and 4 shows the process of segmentation for sample rules R1, R2, R4, R7, and R9 selected from Table 2. Each rectangle represents a rules packet space. White space represents that the action of that rule is “Allow,” and Grey space represents that the action of that rule is “Deny.” Figure 4 shows the final segments formed for the rules considered. Each segment is either a non-overlapping segment or a non-conflicting or conflicting segment.

In this work, a BDD-based segmentation technique proposed in Hu et al. (2012) is used to segment the rules into disjoint segments. This segmentation helps to identify clearly the boundary between intersected and non-intersected packet space. The segmentation process is depicted in Algorithm 1.

A disjoint segment set ${\mathbb{S}} = \{ {\mathfrak{s}}_{1} ,{\mathfrak{s}}_{2} \ldots ,{\mathfrak{s}}_{n} \}$ must satisfy the following two properties:

1. Any pair of segments of ${\mathbb{S}}$ must be disjoint; i.e., ${\mathfrak{s}}_{i} \cap {\mathfrak{s}}_{j} \ne \phi$ for *i*, *j*  $\in$ 1 *ton* and $i \ne j;$
2. Any two packet spaces, $\fancyscript{p} \in {\mathfrak{s}}_{i} ,$ where $\fancyscript{p} \ne \fancyscript{p} ^{\prime }$, must match with an exact set of rules.

#### **Algorithm 1**

Segmentation of given ruleset R into set of disjoint segments S (Hu et al. 2012)
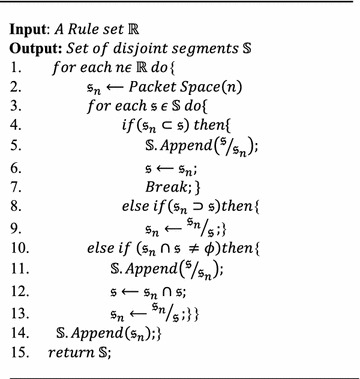


### Anomaly identification using grid representation

In a ruleset, one overlapping segment can be associated with two or more rules, and one rule can be associated with one or more segments. To ease further operation and represent the anomalies more precisely, “grid representation,” that is, a two-dimensional matrix representation, is generated by using ruleset ${\mathbb{R}}$ and set of segments ${\mathbb{S}}$. The Grid representation of the ruleset provided in Table 2 is shown in Table 3.

**Table 3:**
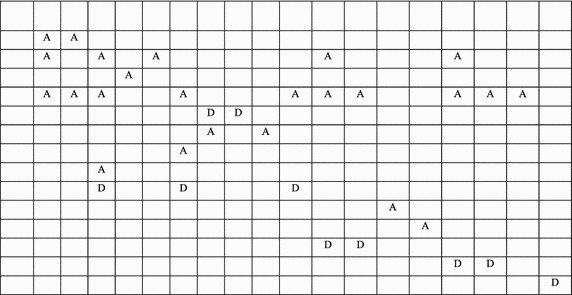
Grid representation of example ruleset of Table 2

In Table 3, “A” indicates that the action is Allow, and “D” indicates that the action is Deny. From the grid representation, one can obtain a clear view of exactly where anomalies are occurring. For example, segments 4, 5, 8, 9, 13, 14, 17 and 18 in Table 3 are non-overlapping segments and all of the remaining segments are overlapping. Among the overlapping segments, 1 and 2 are non-conflicting segments, and 3, 6, 7, 10, 11, 12, 15 and 16 are conflicting segments. An anomaly can be easily identified based on the grid shown in Table 3. For example, rule 9 is shadowed by rule 4 because all of the subspaces covered by rule 9 are also covered by rule 4 but with a different action, and rule 4 contains additional subspaces that are not covered by Rule 9. Therefore, rule 9 is a subset of Rule 4 with a different action and hence *shadowed*. From the grid representation, rule 9 is a *generalized* rule of rule 8 because all of the subspaces covered by rule 8 are also covered by rule 9 but with different actions, and Rule 9 covers additional subspaces that are not covered by rule 8. From the grid, a *correlation* can be identified between rules 5 and 6 because they share a common segment (subspace) s7 having different action. Furthermore, there is a segment covered by rule 5 and not covered by rule 6 and vice versa. *Redundant* rules can also be identified from the grid. For example, “Rule 1” is redundant because, even when rule 1 is removed, the action taken by the firewall for a packet falling in the segment space of rule 1 does not change. However, redundancy depends upon the order of the rules; hence, redundant rules cannot be removed at this stage from the grid but can be removed only after the reordering phase.

**Table 4:** Action constraint generating strategies (Hu et al. 2012)

Strategy	Action constraint
Deny-override	Action = “deny”
Allow-override	Action = “ALLOW”
Recency-override	Action of newest rule
Specificity-override	Action of most-specific rule
High-majority-override	Action of rules with greater number than opposite rules
First-match-override	Action of first-matched rule
High-authority-override	Action of rule with highest authority

## Anomaly resolving

Some earlier works related to “packet-filter firewall policy anomalies,” which addressed the detection stage, and includes the following references (Liang et al. 2014; Hari et al. 2000; Alex 2009; Pozo et al. 2008; Yuan et al. 2006; Benelbahri and Bouhoula 2007; Al-Shaer and Hamed 2004a, b; Muhammad et al. 2006). A few works also addressed the resolving stage. Hari et al. (2000) proposed a “fast detect” framework that was able to detect and resolve correlation conflicts by reordering the rules. However, the authors were only able to address individual local conflicts; they were unable to address global conflicts. The authors used resolve filters to break circular loops formed by rules that could not be resolved by reordering. “Fast detect” is not suitable with present firewalls because it was designed based on two-tuple firewalls. The authors of Muhammad et al. (2006) tried to resolve the conflicts by only following a default deny policy, that is, by denying all of the packets that are in the conflict space. Hu et al. (2012) addressed the issue of both local and global conflict detection and resolution. To resolve the anomalies, “Action Constraints” were generated for the conflicted segments, and reordering of rules was performed based on these action constraints. In the process of identifying the best order of rules, a combination of greedy and permutation algorithms was proposed.

### Action constraint generation

In Hari et al. (2000), the authors defined a semi-automatic mechanism to generate action constraints. The authors introduced a “Risk Level (RL)” to each conflicted segment, which depended upon the “Risk Value.” “Risk Value (RV)” was calculated for every vulnerability in the network using the CVSS (Mell et al. 2007) score and Importance Value (IV) as shown in Eq. 1.

1 $$Risk \,Value \,(RV) = CVSS(v) \times IV(s)$$

The RL for a conflicted segment is calculated as shown in Eq. 2, which is nothing but the accumulated RVs of vulnerabilities belonging to that segment.

2 $$RL_{CS} = \frac{{\mathop \sum \nolimits_{v \in V(CS)} RV}}{{\alpha \times \left| {V(CS)} \right|}}$$

where *CS* denotes conflicting segment, *V*(*CS*) is set of Vulnerabilities in *CS*, $CVSS(v)$ is CVSS of Vulnerability $v$, $IV(s)$ is IV of a Service “S,” and $\alpha$ is a factor assigned by the administrator to control the dependency of RL on the vulnerabilities in CS. The administrator also assigns IV to a service based on the importance of the service.

The CVSS is an open framework designed for calculation of the Risk involved in an organization. CVSS uses several metrics as discussed in Mell et al. (2007) for calculating vulnerability score. There are different strategies for generating action constraints as proposed by Hu et al. (2012), which are shown in Table 4. The administrator can define customized threshold values of RL called upper limit (UL) and lower limit (LL). For a conflicted segment, if the obtained RL is greater than UL or less than LL, then the action constraint is generated automatically. The action constraint follows an automatic “Deny-Override” strategy when the RL is greater than the UL and an “Allow-Override” strategy if RL is less than the LL. However, when the RL is in-between LL and UL, a manual strategy selection is used. This study proposes an automatic action-constraint strategy.

### Rule reordering and redundancy removal

Once Segmentation, Conflict Group Formation, and Action Constraint Generation is performed, based on the results, the administrator can manually change the order of the rules to eliminate conflicts. However, changing the order of the rules manually will be a tedious task when the size of the policy is huge. Therefore, an automated mechanism is to be used to reorder the rules by segment sets and their action constraints. The authors in Hu et al. (2012) used a combination of greedy and permutation to find the best possible order to resolve the conflicts. However, there is a limitation in this approach; an optimal solution might not be obtained in all of the cases. This study proposes to use ACO to find the best possible order of rules, which can produce more-optimal results. FAME (Hu et al. 2012) also proposed a separate method to remove redundancy. However, it is a semi-automated approach. In our work, redundancy is automatically addressed by the ACO itself without the need of a separate process.

## ACOFAME

This work proposes adaptive and automated detection and resolution of firewall policy anomalies. The proposed modifications include the following:

- The “Action Constraint Generation” is modified to include a *TF* for a source IP address to establish a relationship between security policy and the resolver, which increases the chance of security policy conformation after resolving and reordering.
- “Action Constraint Generation” is automated.
- ACO is used to address reordering and redundancy removal without the need for an additional redundancy removal phase.
- Anomaly detection and resolution for a newly added rule can be quickly performed adaptively, which can save much time compared with running the tool for *n* + 1 rules.

### “Trust Factor”-based and automated “Action Constraint Generation”

**Fig. 5:**
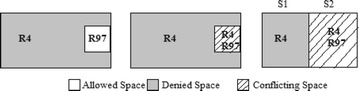
Segmentation of rule 4 and rule 97

**Fig. 6:**
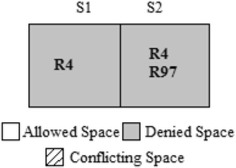
Segmentation after action constraint generation based on FAME

The authors in Hu et al. (2012) used the CVSS score and IV for calculating RL of a conflicting segment. The CVSS score and IV are both defined from the organization’s asset, i.e., only the destination address of a packet. Considering destination system parameters alone when generating “Action Constraints” will increase the availability loss, which indicates that the firewall will drop packets that are supposed to be allowed. Assume that the host “192.168.124.125” is an internal server with a high-RV, for example, 9 out of 10. An administrator adds Rule 97 to provide remote access to trusted host “172.19.23.22” without knowing about rule 4 as shown in Table 5. An anomaly exists; rule 97 is shadowed by rule 4; hence, remote access from the trusted host will be denied. Figure 5 shows the segments formed by the two Rules 4 and 97. There exists a conflict as shown in segment 2. Because the Risk calculation is based only on the destination address, although the administrator trusts the source host, his intention cannot be satisfied. Segment 2 denies the packet space as shown in Fig. 6 based on the action constraint generated by FAME.

**Table 5:** Sample ruleset

Rule	Protocol	Source IP	Source port	Destination IP	Destination port	Action
4	*	*	*	192.168.124.125	*	Deny
.	…	…	…	…	…	…
.	…	…	…	…	…	…
97	TCP	172.19.23.22	*	192.168.124.125	22	Allow

Based on the security policy, a *TF* is allocated to all of the sources that are trusted by the administrator to obtain the intended behavior from the firewall. TF is used as one of the parameters in computing the new RL of a conflicted segment $RL_{CS}^{\prime }$ as shown in Eq. 3. The TF can be between 0 and 1; “1” indicates a highly trusted source, and “0” indicates an untrusted source.

3 $$RL_{CS}^{\prime } = \frac{{\mathop \sum \nolimits_{{\left( {{\mathfrak{a}}_\mathfrak{S} ,{\mathfrak{a}}_\fancyscript{d} } \right) \in CS}} \left( {RV^{\prime } \left( {{\mathfrak{a}}_\fancyscript{d} } \right) \times \left( {1 - TF\left( {{\mathfrak{a}}_{\mathfrak{S}} } \right)} \right)} \right)}}{N(CS)}$$

where *CS* is conflicting segment, *N*(*CS*) is Number of $({\mathfrak{a}}_{\mathfrak{S}} ,{\mathfrak{a}}_\fancyscript{d} ),$ source and destination pairs, that belongs to *CS*, $TF({\mathfrak{a}}_{\mathfrak{S}})$ is TF of Source a_s_, and $RV^{\prime } ({\mathfrak{a}}_\fancyscript{d})$ is total risk associated with Destination a_d_ as follows:

4 $$RV^{\prime } ({\mathfrak{a}}_{\fancyscript{d}} ) = \frac{{\mathop \sum \nolimits_{{v \in V\left( { {\mathfrak{a}}_{\fancyscript{d}} } \right)}} RV}}{{\left| {V\left( {CS} \right)} \right|}}$$

This work proposes a new and automated algorithm to generate action constraints that use five of the strategies shown in Table 4. The associated pseudocode is shown in Algorithm 2.
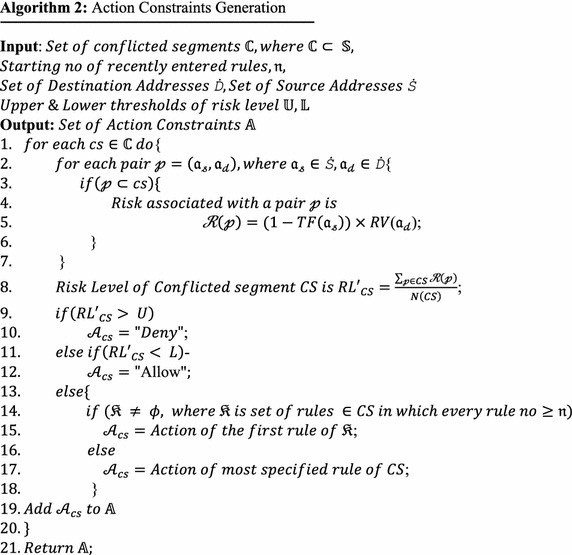


The Modified RVs $(RV^{\prime } )$ shown in Table 6 are calculated using Eq. 4, which uses Risk Value *RV*. The *RV* is calculated using Eq. 1, uses the CVSS framework suggested by Mell et al. (2007) and Hu et al. (2012). Table 7 shows that the *TFs* assigned by the administrator for the scenario depicted in Fig. 1 are deduced from the security policy of Table 1.

**Table 6:** List of organization IT assets and their risk values

Destination IP address	
10.12.32.21	4
10.12.32.22	6
10.12.32.23	10
10.12.32.24	9
10.12.32.25	10
10.12.32.26	10

**Table 7:** List of source addresses and their trust factors

Source IP address	
192.168.5.64	0.9
192.168.15.253	0.9
10.44.128.112	0.9
172.19.55.121	0.6
172.19.55.124	0.6
172.19.55.122	0.1
172.19.55.123	0.1
10.45.48.34	0.1
172.19.64.221	0

Five strategies from among those suggested in Table 4 were used to automate action constraint generation completely as shown in Algorithm 2. When the RL is above the higher level, “Deny-Override” is chosen as the “Action Constraint.” When the RL is below the lower level, “Allow-Override” is chosen. Otherwise, Recency-Override with First-Match Override will come into effect. Therefore, the action of the first rule among the recent ones in that conflicted segment will be chosen. Here, recency number ${\mathfrak{n}}$ is automatically obtained from the parameter “Recency Interval,” which will be set by the administrator once as part of the organization’s policy before running ACOFAME. The value ${\mathfrak{n}}$ is the serial number of the first rule among the set of recent rules. The recency strategy helps to decide the action to be taken in case of conflict. Whenever there is a new security need, the administrator will add a new rule based on that need, but there is a chance that the new rule might come into conflict with the earlier rules. In such a situation, the newly entered rules obviously must take priority over the earlier rules. If there are no such rules that fall under the recent rules, then specificity-override will be chosen. Therefore, the action selected for that conflicted segment is that of the most specific rule among the rules covered in that segment. These strategies were proved promising with the help of result analysis, which will be discussed later.

**Fig. 7:**
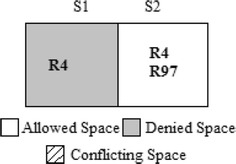
Segmentation after action constraint generation based on proposed algorithm

By employing the proposed *TF*-based action constraint generation for the example scenario shown in Table 5, if the TF assigned to the trusted source IP of rule 97 is 1, then the new Risk Level *RL*′ for the conflicted segment s2 will be 0. Therefore, from the proposed algorithm, the action constraint for segment s2 will be “Allow” as shown in Fig. 7. The administrator’s wish to allow a trusted host “172.19.23.22” of rule 97 is now satisfied, and the availability loss is reduced. Table 8 shows the action constraint generated by the proposed algorithm for the grid shown in Table 3. The last row of Table 8 shows the Strategies applied by Algorithm 2 for the given conflicted segments. The ‘–‘indicates that there is no conflict in that segment.

**Table 8:** Action constraints generated by proposed approach for the grid shown in Table 3

Segments																		
Action constraints	A	A	A	A	A	A	A	D	A	D	A	D	A	A	A	D	A	D
Strategy applied by Algorithm 2	–	–	AO	–	–	AO	AO	–	–	DO	AO	RFMO	–	–	AO	RFMO	–	–

*AO* allow override, *DO* deny override, *RFMO* recency with first match override

### Motivation for applying ACO

The ACO algorithm is a metaheuristic (Dorigo and Caro 1999)-based optimization approach designed based on a biological ant system. The Swarm Intelligence approach is inspired by nature (Bonabeau et al. 1999). It primarily uses two factors, i.e., the pheromone and heuristic factors, as an aid in finding a solution. It was initially introduced by Dorigo et al. (1991, 1996), Dorigo (1992) at the beginning of the 1990s. They used ACO to find an optimal path from a source to a destination through a group of nodes connected by multiple paths. The authors in Dorigo and Caro (1997) used ACO to solve the Traveling Salesman Problem (TSP), which is to find the best order of cities to travel to, minimize the total distance traveled. The distance between pairs of cities is used as a heuristic factor. To work with ACO, one must convert the given problem into a two-dimensional mesh. The authors in Liangjun et al. (2008), Jensen and Shen (2003), Majdi and Derar (2013) proposed a Rough set attribute reduction using an ACO-based approach to reducing the number of attributes in a dataset. They used Rough set Significance as a heuristic factor that was calculated when needed. The authors of Ravi Kiran Varma et al. (2015) proposed a novel ACO search for global best attributes by considering Rough Set-based attribute significance as the heuristic factor for Ant search. Apart from these examples, ACO is also used in solving many NP-Hard and combinatorial optimization problems. Some of these include vehicle traffic (Jabbarpour et al. 2014), University course timetabling (Socha et al. 2002), Multicast Routing (Zhang and Liu 2011), Stock Market Prediction (Binoy et al. 2011), and Bankruptcy Prediction (Nigib et al. 2013). The authors of Broderick et al. (2014) employed ACO to solve a Software Project Scheduling problem. The heuristic factor changes based upon the type of problem. ACO was also used for feature selection in the signal-processing domain in Turker et al. (2014). The work in Sreelaja and Vijayalakshmi (2010) proposed an ACO-based packet-filter firewall to overcome the drawbacks of the Neural Network, Binary search, and sequential search approaches.

**Fig. 8:**
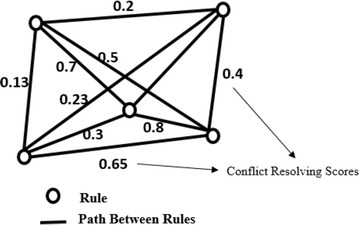
Sample ACO Graph of proposed approach

This work proposes an ACO-based reordering algorithm by taking conflict resolving score (CRS) as a heuristic factor, which can be calculated as and when needed. The problem can be converted into a graph as shown in Fig. 8, in which each node is nothing but the firewall rule.

After converting the problem into a two-dimensional mesh network, artificial ants are released at random nodes. The ants will traverse from one node to another by selecting next node at each step using probability calculated from two factors that are a heuristic factor and pheromone factor using Eq. 5 (Dorigo and Thomas 2004).

5 $$p_{ij}^{k} = \left\{ {\begin{array}{*{20}l} {\frac{{\tau_{ij}^{\alpha } \times \eta_{ij}^{\beta } }}{{\mathop \sum \nolimits_{{l \in N_{i}^{k} }} \tau_{il}^{\alpha } \times \eta_{il}^{\beta } }}} \hfill &\quad {if\, j \in N_{i}^{k} } \hfill \\ 0 \hfill &\quad { otherwise} \hfill \\ \end{array} } \right.$$

*i* is present node, *j* is next node, $\tau_{ij}$ is pheromone concentration on branch *ij*, $\eta_{ij}$ is heuristic factor calculated for branch *ij*, and $N_{i}^{k}$ is remaining nodes that are not traversed by ant k.

The heuristic factor depends upon the type of problem. For example, for the TSP, the heuristic factor is calculated using the formula shown in Eq. 6.

6 $$\eta_{ij} = 1/d_{{ij}}$$

where *d*_*ij*_ is the distance between *i* and *j*.

The parameters $\alpha$ and $\beta$ are used to control the importance of heuristic and pheromone factors. If $\alpha = 0,$ the ants will select the next city closest to them, which turns ACO into simple greedy algorithms. If $\beta = 0,$ only pheromone amplification will occur. Initially, the pheromone concentration will be same on all branches and hence in the first iteration the movement of ants completely depends upon the heuristic factor. After every iteration, the pheromone is updated on the branches that are traversed and evaporated on all remaining branches that are not traversed. From the second iteration onwards, because of the varied pheromone concentration, the ants will start converging toward an optimal path, giving a common optimal solution after several iterations. The formula used to update and evaporate pheromones is shown in Eqs. 7 and 8, respectively.

7 $$\tau_{ij} = (1 - \rho ) \cdot \tau_{ij} + \mathop \sum \limits_{k = 1}^{m} \Delta \tau_{ij}^{k}$$

8 $$\Delta \tau_{ij}^{k} = \left\{ {\begin{array}{*{20}l} {\frac{q}{{L_{k} }} } \hfill &\quad {if\, ant\, k\, used\,edge\, ij\, in\, the\, tour} \hfill \\ 0 \hfill &\quad {otherwise} \hfill \\ \end{array} } \right.$$

*L*_*k*_ is the total distance of the path traversed by ant *k*, and $\rho$ is the evaporation rate.

### ACO heuristic for the problem domain and the resolver algorithm

After determining the action constraints, the goal is to find the best possible ordered combination of rules that produces the highest *CRS*. The CRS is nothing but the count of segments that satisfy the action constraints, which will be used in calculating heuristics for ACO as shown in Eq. 9. A segment is considered satisfied if the action constraint generated for that segment and the action of the first rule under that segment are the same. Otherwise, the segment is considered not satisfied.

For example, in the grid representation shown in Table 3, the action constraint generated for segment 7 is “ALLOW,” which was shown in Table 8. For the initial order of rules shown in Table 3, segment 7 is considered not satisfied because the first vertical subspace under that segment 7 is Rule 5, and its action is “DENY.” The CRS for this initial order is 14, as shown in Table 9, because 14 segments were satisfied and 4 segments were unsatisfied. The heuristic factor $\eta$ used for this problem domain is shown in Eq. 9:

9 $$\eta_{\fancyscript{i}\fancyscript{j}}^{k} = \frac{{CRS\left( {\fancyscript{t}_\fancyscript{k} \cup \fancyscript{j}} \right) - CRS\left( \fancyscript{t}_\fancyscript{k} \right)}}{{Segments\, covered\, by\, (\fancyscript{t}_{k} \cup \fancyscript{j})}}$$

$$\fancyscript{t}_{k}$$ is the set of rules traversed by ant $$k$$, $$\fancyscript{i}$$ is the last rule of $$\fancyscript{t}_{k} ,$$$$\fancyscript{j}$$ is the next rule to be traversed, and $$CRS(\fancyscript{t}_{k} ),$$ the number of segments satisfied, is calculated using Eq. 10:

10 $$CRS(\fancyscript{t}_{k} ) = \left| {{\mathbb{S}}_{{\fancyscript{t}_{k} }} } \right|$$

${\mathbb{S}}_{{\fancyscript{t}_{k} }}$ is the set of satisfied segments associated with ${\fancyscript{t}}_{k}$

**Table 9:** An example for CRS calculation for the order of rules 1–14 (Policy 1) of Table 2

Segments																		
Action constraints generated by Algorithm 2 for Policy 1	A	A	A	A	A	A	A	D	A	D	A	D	A	A	A	D	A	D
Action in first vertically appearing block in the segments of Table 4	A	A	A	A	A	A	D	D	A	A	A	A	A	A	A	A	A	D
Satisfied (S)/not satisfied (NS)	S	S	S	S	S	S	NS	S	S	NS	S	NS	S	S	S	NS	S	S

*A* allow, *D* deny

### ACO algorithm applied to reorder the rules to resolve anomalies

The rules are copied into ${\mathbb{R}}^{\prime }$ from ${\mathbb{R}}$ according to the output of Algorithm 3.
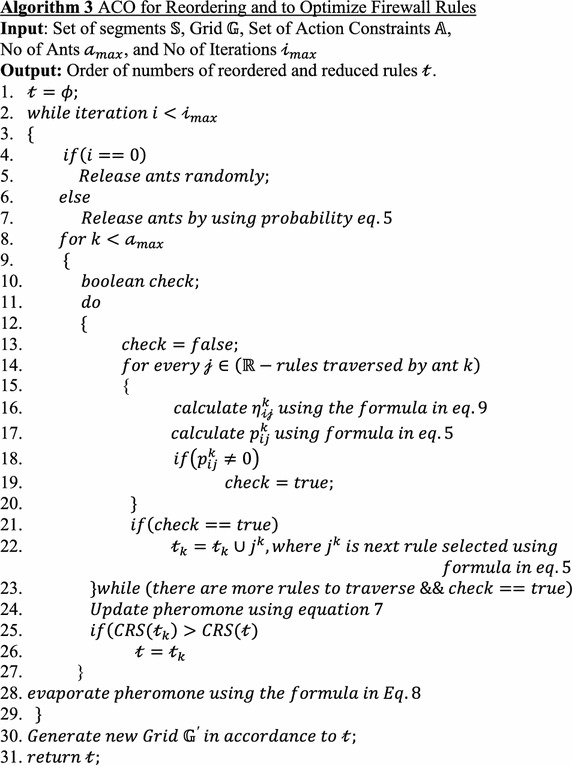


Algorithm 3 shows the rule reordering process. $\fancyscript{i}_{max }$ number of ants are released in each iteration for $\fancyscript{a}_{max}$ iterations. In each iteration, every ant will find its own solution. For this problem, each solution is nothing but a possible order of rules. For the first iteration, ants are released randomly because there will not be any influence of pheromone in the first iteration. Every ant finds a solution, an order of the rules by traversing each rule based on the selection probability. In this algorithm, the pheromone is updated for every ant’s solution so that the pheromone concentration increases toward the possible best solution. For each iteration, the iteration’s best ant solution is saved, which is nothing but the solution with the highest CRS. The pheromone is evaporated at the end of each iteration. Finally, after the last iteration, the global best solution is determined.

### Adaptive detection and resolution

This work also proposes a mechanism that adaptively reorders the existing rules when an administrator enters a new rule even after running the ACOFAME tool. The advantage is that much time will be saved when compared with running the tool for n + 1 rules again. The algorithm for adaptive reordering is shown in Algorithm 4. The input for this algorithm is the resolved and reordered ruleset after applying ACOFAME. When a new rule is entered by the administrator, it will be compared against the existing reordered ruleset ${\mathbb{R}}^{\prime }$ one by one, sequentially. The following are the possible situations.

#### *Case 1: Subset and different action*

If the new rule $r$ is a subset of the compared rule and the action of the compared rule and the new rule are not same. In this case, to make an appropriate decision concerning whether to “Allow” or “Deny,” an Action Constraint will be generated using Algorithm 2. If the generated action constraint is same as that of the new rule, then the new rule is inserted before the compared rule position in the reordered ruleset and the reordered rule grid is updated. Conversely, if the generated Action Constraint is different, then obviously the new rule will be ignored.

#### *Case 2: Subset and same action*

If the new rule $r$ is a subset of the compared rule and the action of the compared rule and the new rule are same. In this case, the new rule will be a shadowed rule and redundant, and hence ignored.

#### *Case 3: Intersection and different action*

If there exists an intersection or correlation among the new rule $r$ and the compared rule, and the action of the compared rule and the new rule are not same. In this case, to make an appropriate decision concerning whether to “Allow” or “Deny,” an Action Constraint will be generated using Algorithm 2. If the generated action constraint is same as that of the new rule, then the new rule is inserted before the compared rule position in the reordered ruleset and the reordered rule grid is updated.

#### *Case 4: Intersection and same action*

In this case, the new rule will not be inserted. The “if” condition of line number 11 of algorithm 4 will fail, and the new rule is sequentially checked with the remaining rules. Finally, if none of the cases match, then the new rule is appended to the existing reordered ruleset.
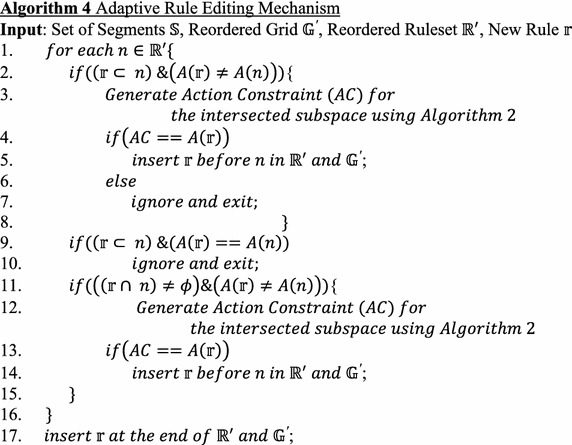


## Experimentation results and comparison

### Lab environment and datasets

The experiments were performed on an Intel Core 2 Duo CPU 2.6 GHz with 2 GB RAM and the Windows 7 Operating System. The free parameters used in the ACO are set to the following values on a trial and error basis, and this set of parameters are found to be suitable: $\alpha = 1$, $\beta = 0.01$, $\rho = 0.9$, and *q* = 0.9. The initial value of the pheromone is set to 0.5. As of now, there are no standard benchmark packet-filter firewall rules available. Five rulesets were used, named Policy 1–5, as shown in Table 10. The rulesets were collected from college and university level campus networks. The number of anomalies identified in each dataset is shown category-wise in Table 10. All the algorithms listed in this paper were developed using Java SE 1.7. Java with jpcap-0.6 was used for testing and simulation of firewall. *Nping*, network packer generation tool, was used to generate network traffic for testing. Nessus vulnerability scanner was used in the process of identifying vulnerabilities associated with the systems that belongs to the conflicted segments.

**Table 10:** Policy-wise segments and number of anomalies

Policy no.	No of rules	No. of segments generated after segmentation	Time taken (s)	No of shadow anomalies	No of generalization anomalies	No of correlation anomalies	No of redundant rules
Policy 1	14	18	0.016	3	1	4	2
Policy 2	35	35	0.013	7	6	3	4
Policy 3	55	64	0.064	9	8	6	6
Policy 4	171	171	0.482	29	29	8	35
Policy 5	325	344	0.846	45	54	20	49

### Resolved and reordered output of ACOFAME

The policies, the number of rules, the number of segments generated by the segmentation process and the time taken for segmentation are shown in Table 10 along with categories of anomalies found in each ruleset. The rules for Policy 1 are nothing but the rules shown in Table 2. The proposed ACOFAME algorithm was used to reorder the rules for all of the policies. The results of reordered rules and the reordered grid are shown in Tables 11 and 12, respectively.

**Table 11:** Reordered output of ACOFAME (input: Policy 1)

Rule	Protocol	Source IP	Source port	Destination IP	Destination port	Action
1	TCP	10.44.128.112	*	10.12.32.23	23	Allow
2	*	10.*.*.*	*	10.12.32.21	80	Allow
3	*	10.45.48.34	*	10.12.32.*	*	Deny
4	TCP	192.168.5.64	*	10.12.32.23	23	Allow
5	TCP	172.19.55.*	*	10.12.32.21	80	Allow
6	*	10.*.*.*	*	10.12.32.21	80	Allow
7	*	172.19.55.123	*	10.12.32.*	*	Deny
8	TCP	192.168.15.253	*	10.12.32.23	23	Allow
9	TCP	172.19.55.121	*	10.12.32.24	20–21	Allow
10	*	172.19.55.121	*	10.12.32.*	*	Deny
11	*	172.19.55.121–172.19.55.124	*	10.12.32.*	*	Allow
12	*	172.19.64.221	*	10.12.32.*	*	Deny

**Table 12:** Grid with reordered rules resolved by ACO

S. no.	Order																		
1															A				
2								A		A									
3								D	D										
4					A														
5		A		A		A						A				A			
6												D	D						
7																D	D		
8														A					
9							A												
10				D			D				D								
11		A	A	A			A				A	A	A			A	A	A	
12																			D

### Performance evaluation parameters and comparison

The parameters that are used for evaluation and comparison of the proposed system are *availability loss, security risk, number of resolved conflicts, number of redundant rules eliminated,* and *the time taken by the algorithm.*

#### Availability loss

*Availability loss (AL)* Hu et al. (2012) is used to measure the effect of a firewall policy on network availability. Availability loss occurs if the action constraint generated for a conflicted segment is “ALLOW,” but the action taken by the firewall on that packet is “DENY.”

AL for a particular firewall policy ruleset ${\mathcal{P}}$, is calculated using the formula shown in Eq. 11, which is a modified version of the availability loss equation proposed in Hu et al. (2012).

11 $${\mathcal{A}}\ell \left( {\mathcal{P}} \right) = \mathop \sum \limits_{{cs \in CS\left( {\mathcal{P}} \right) \cdot is\,Forced\,Denied}} \left( {10 - RL^{\prime } \left( {cs} \right)} \right)$$

*An example calculation of Availability Loss for the ruleset shown in Policy 1*$\text{ }({\mathcal{P}}1)$:

To calculate the AL of Policy 1, the conflicted segments of Policy 1 before reordering (Table 3) and the action constraints generated for those segments (Table 9) must be observed. Segment 7 $(S_{7} )$ was the only segment in which the generated action constraint was Allow but the performed action was Deny. In Segment 7, there is a conflict between rule 5 and rule 6. The source IP of the conflicted segment is “10.45.48.34,” and the destination IP of the conflicted segment is “10.12.32.21.” Therefore, the source, “10.45.48.34,” will not obtain web access to the web server; hence, availability loss occurs. From Eq. 3,

$$\begin{aligned} RL^{\prime } \left( {S7} \right) & = RV^{\prime } (10.12.32.21) (1 - TF(10.45.48.34))/1 \\ & = 4\left( {1 - 0.1} \right)/1 = 3.6 \\ \end{aligned}$$

Substituting $RL^{\prime } (S7)$ in Eq. 11,

$$\begin{aligned} {\mathcal{A}}\ell \left( {{\mathcal{P}}1} \right) & = \mathop \sum \limits_{{cs \in \{ S7\} }} \left( {10 - RL^{\prime } \left( {cs} \right)} \right) \\ & = 10 - RL^{\prime } \left( {S7} \right) = 10 - 3.6 = 6.4 \\ \end{aligned}$$

Availability Loss was calculated similarly for other rulesets (Policies 2–5). Note that the Availability Loss after the reordering of Policy 1 with our proposed method is 0 because, after reordering Policy 1, the action taken by the firewall on conflicted segment S7 is “Allow,” and the generated action constraint is also “Allow.”

#### Security risk

*Security Risk* This parameter is helpful to measure the security risk to the organization’s network due to the firewall policy. It is calculated as shown in Eq. 12, which is a modified version of the security risk equation proposed in Hu et al. (2012).

12 $${\mathcal{S}}\fancyscript{r}\left( {\mathcal{P}} \right) = \mathop \sum \limits_{{cs \in CS({\mathcal{P}}) \cdot is\,Allowed}} \left( {RL^{\prime } (cs)} \right)$$

*An example calculation of Security Risk for the ruleset shown in Policy 1*$\text{ }({\mathcal{P}}1)$:

Conflicting segments whose first vertical action in the segments is “Allow” must be identified. From Table 3, Policy 1 has 7 conflicting segments. These segments are

$$CS\left( {{\mathcal{P}}1} \right) = \left\{ {S3, \, S6, \, S10, \, S11, \, S12, \, S15, \, S16} \right\}$$

For each segment of ${\text{CS}}({\mathcal{P}}1),$ we must calculate ${\text{RL}}^{\prime } ({\text{cs}})$. A sample calculation for ${\text{RL}}^{\prime } ({\text{S}}3)$ is shown:

The packet space covered by Segment 3 is

“* 172.19.55.121 * 10.12.32.23–10.12.32.26 *”

In this packet space, there is one source, ${\mathfrak{a}}_{S1}$, which is “172.19.55.121,” and four destinations, ${\mathfrak{a}}_{\fancyscript{d}1} \,to\,{\mathfrak{a}}_{\fancyscript{d}4}$, which are “10.12.32.23–10.12.32.26.” Therefore, the number of source–destination pairs is 4. Calculation of $RL^{\prime }$ for conflicted segment 3 (s3) using Eq. 3 is shown as an example.

$$RL_{S3}^{\prime } = \frac{{\begin{array}{*{20}c} {\left( {RV^{\prime } \left( {{\mathfrak{a}}_{\fancyscript{d}1}} \right) \times \left( {1 - TF\left( {{\mathfrak{a}}_{\fancyscript{S}1}} \right)} \right)} \right) + \left( {RV^{\prime } \left( {{\mathfrak{a}}_{\fancyscript{d}2 }} \right) \times \left( {1 - TF\left( {{\mathfrak{a}}_{{\fancyscript{S}}1} } \right)} \right)} \right)} \\ { + \left( {RV^{\prime } \left( {{\mathfrak{a}}_{\fancyscript{d}3} } \right) \times \left( {1 - TF\left( {{\mathfrak{a}}_{\fancyscript{\fancyscript{S}}1} } \right)} \right)} \right) + \left( {RV^{\prime } \left( {{\mathfrak{a}}_{\fancyscript{d}4} } \right) \times \left( {1 - TF\left( {{\mathfrak{a}}_{\fancyscript{S}1} } \right)} \right)} \right)} \\ \end{array} }}{4}$$

By substituting pre-calculated values of $RV^{\prime }$ from Table 6 and TF values from Table 7,

$$\begin{aligned} & = \frac{{\left( {10 \times \left( {1 - 0.6} \right)} \right) + \left( {9 \times \left( {1 - 0.6} \right)} \right) + \left( {10 \times \left( {1 - 0.6} \right)} \right) + \left( {10 \times \left( {1 - 0.6} \right)} \right)}}{1 \times 4 = 4} \\ & = \frac{15.6}{4} = 3.9 \\ {\mathcal{S}}\fancyscript{r}\left( {{\mathcal{P}}1} \right)& = \mathop \sum \limits_{{cs \in CS\left( {\mathcal{P}} \right) \cdot is\,Allowed}} \left( {RL^{\prime } \left( {cs} \right)} \right) \\ & = RL^{\prime } \left( {S3} \right) + RL^{\prime } \left( {S6} \right) + RL^{\prime } \left( {S10} \right) + RL^{\prime } \left( {S11} \right) + RL^{\prime } \left( {12} \right) + RL^{\prime } \left( {15} \right) + RL^{\prime } \left( {16} \right) \\ & = 3.9 + 1.8 + 7.6 + 2.5 + 4.9 + 2.8 + 4.5 = 28 \\ \end{aligned}$$

The *Security Risks* of other rulesets are calculated similarly.

#### Comparison of evaluation parameters

To prove the influence and importance of TF in reducing Availability Loss, the ALs of reordered policies proposed by the FAME method and our method are calculated and compared. The ALs of policies reordered with the help of the action-constraint generation methodology of FAME are provided in column 5 of Table 13. The policies show a certain amount of Availability Loss even after reordering, whereas the ALs of policies reordered with the help of action constraints generated based on TF are 0. Table 13 also shows the total number of segments generated, number of conflicted segments, and time taken for action constraint generation. It is clear that there is no availability loss with our approach.

**Table 13:** Comparison of availability loss after reordering of policy rules with action constraints generated by FAME and ACOFAME

Policy no.	Total no. of segments generated	No. of conflicting segments	Time taken for action constraint generation (in music)	Availability loss (with action constraints generated by FAME)	Availability loss (with action constraints generated by ACOFAME)
Policy 1	18	8	<1	18	0
Policy 2	35	19	2	35	0
Policy 3	64	33	2	64	0
Policy 4	171	77	7	171	0
Policy 5	344	164	59	344	0

Table 14 shows the experimental results for all of the five policy rule sets. The second column depicts the actual conflicts existing in each policy. The third column shows the number of Resolved Conflicts (RC) and time taken to resolve the conflicts by the Permutation method proposed by Hu et al. (2012). The fourth column shows the number of RC and time taken to resolve the conflicts by the Greedy method proposed by Hu et al. (2012). The fifth column shows the number of RC and time taken to resolve the conflicts by the Combination method proposed by Hu et al. (2012). The sixth column shows the number of RC, the time taken to resolve the conflicts, and the number of Rules Eliminated (RE) automatically due to redundancy by the ACOFAME. The seventh and last column shows the percentage of conflicts resolved and clearly indicates that ACOFAME has outperformed FAME with respect to conflicts resolved. The values in the italics indicates improvement in the results. One advantage of ACOFAME is that the redundancy removal and anomaly resolving is performed simultaneously as opposed to with the FAME method, in which each is a different phase.

**Table 14:** Evaluation of ACOFAME and comparison with FAME

Policy no.	No. of conflicted segments	Permutation (Hu et al. 2012)		Greedy (Hu et al. 2012)		Combination (FAME) (Hu et al. 2012)		ACOFAME (proposed work)			% of conflicts resolved	
		RC	Time (s)	RC	Time (s)	RC	Time (s)	RC	Time (s)	RE	FAME	ACOFAME
1	8	8	0.142	3	0.016	8	0.128	8	0.196	2	100	100
2	19	19	0.229	13	0.018	19	0.178	19	0.918	4	100	100
3	33	33	31.215	25	0.040	31	0.589	*33*	4.447	6	93.93	*100*
4	77	–	∞	63	0.046	71	25.281	*77*	122.28	35	92.92	*100*
5	164	–	∞	126	0.048	152	32.722	*163*	1754.12	49	92.68	*99.39*

The results shown in Table 14 indicate that ACOFAME can resolve more conflicts than can the other approaches. ACOFAME requires less time to resolve than the permutation approach does, but more time than do the greedy and FAME approaches. Although ACOFAME requires more time to resolve the conflicts than FAME does, this drawback can be neglected because the Availability Loss and Security Risk are the least compared with other approaches.

**Fig. 9:**
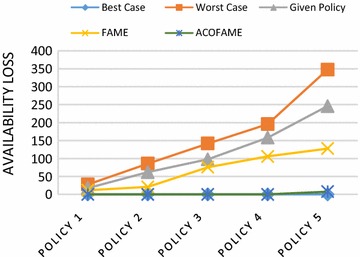
Availability loss evaluation

Figure 9 is the comparative graph showing the *Availability Loss* for each case. Best Case is true when the action performed by the firewall is the same as the organization’s intended action in the respective conflicted segments. Worst Case is true when the firewall “Denies” all of the packets that fall under the conflicted segments. Given Policy is the availability loss calculated for the policy under consideration before reordering. FAME is the availability loss obtained by the FAME algorithm (Hu et al. 2012). ACOFAME is the proposed work; the proposed approach is very close to the Best Case, and the Availability Loss is much less than with existing approaches.

**Fig. 10:**
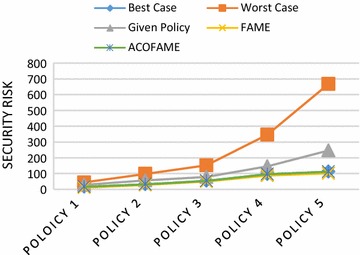
Security risk

Figure 10 is the comparative graph showing the *Security Risk* for each case. Best Case is the same as in Availability Lost, However, in calculating Security Risk, Worst Case is true when the firewall “ALLOWS” all of the packets that fall under the conflicted segments. The Security Risk was reduced compared with the Given Policy, which is nothing but the original policy before reordering. However, the Security Risk is slightly higher compared with FAME because FAME will DENY packets in cases in which the organization’s/administrator’s intention is to ALLOW, as discussed with an example in “ACOFAME” section.

### Advantage of adaptive reordering when a new rule is appended

To evaluate the adaptive rule editing mechanism, the time taken to check one rule adaptively is compared with the time taken by ACOFAME to recheck the entire policy with *n* + 1 *rules*, as shown in Table 15. The time taken for checking a rule by our adaptive mechanism is much smaller than the time taken for rechecking the entire policy with n + 1 rules.

**Table 15:** Advantage of adaptive rule reordering mechanism

Policy no.	Time taken for adaptive checking one rule (s)	Time taken by ACO to recheck *n* + 1 *rules* (s)
Policy 1	0.002	0.198
Policy 2	0.002	0.987
Policy 3	0.004	4.620
Policy 4	0.006	118.57
Policy 5	0.007	1853.44

### Case study 1: Comparison of ACOFAME with Saadaoui et al. (2014)

#### Results when ACOFAME applied to the ruleset data of Saadaoui et al. (2014)

As a case study, our approach is also compared with a recent similar work proposed in Saadaoui et al. (2014). The authors Saadaoui et al. (2014) suggested novel methods for anomaly detection and resolution. They used a two-level approach. In level one, superfluous anomalies were eliminated, and in level two, conflicting rule-class anomalies were eliminated. However, the security policy was verified only in level two, i.e., for conflicting class anomalies, whereas the proposed ACOFAME builds a relationship to the security policy and the conflict resolver through RVs and TFs.

Experiments were conducted to compare our approach with the second topology shown in Fig. 9 of Saadaoui et al. (2014), for which both input and output rulesets are available for comparison. The ruleset related to that topology is shown in Table 16. The implementation was done on the Java platform.

**Table 16:** Ruleset of Saadaoui et al. (2014)

Rule no.	Action	Protocol	Port no.	SIP	DIP
R1	Accept	TCP	80	10.0.0.0/16	172.16.0.22/30
R2	Accept	TCP	80	10.1.0.0/16	172.16.0.22/30
R3	Deny	TCP	80	192.168.0.0/23	172.16.0.22/30
R4	Deny	TCP	80	10.0.0.0/15	172.16.0.22/30
R5	Deny	TCP	80	192.168.0.0/24	172.16.0.22/30
R6	Deny	TCP	80	192.168.1.0/24	172.16.0.22/30
R7	Deny	TCP	80	10.0.0.1	172.16.0.0/16
R8	Deny	TCP	*	10.0.0.1	172.16.0.22/30
R9	Accept	TCP	80	192.168.0.0/24	172.16.0.0/16

Based on the security policy given and the topology, RVs for all of the destinations were calculated based on the framework discussed in ““Trust Factor”-based and automated “Action Constraint Generation”” section. This framework is a modified version of the CVSS framework discussed in “Action constraint generation” section. A few assumptions were made in calculating the RVs of all of the subzones presented in Fig. 9 of Saadaoui et al. (2014) because we cannot obtain all of the details of the parameters required to calculate RV. However, the assumptions are valid because the security policy and the administrator’s experience are considered. TFs are also assigned to all of the subzones of the second topology provided in Fig. 9 of Saadaoui et al. (2014) based on the security policy they provided. Any subzone can be either source or destination. TFs are assigned to sources as shown in Table 17, and RVs are assigned as shown in Table 18 to destinations. A TF of 0.4 (anything less than half) is assigned to 10.0.0.1 and 192.168.0.0/24 because they were mentioned in the security policy and were denied access to subzone31. Other sources are assigned 0 because there are no data available regarding these sources in the security policy. Concerning RVs, subzone31 was mentioned in the security policy. Because servers or systems in that zone are typically associated with high risk, 8 was assigned. For all other destinations, an average risk of 5 is assumed. Table 19 shows the output of ACOFAME, and it can be verified that the output also conforms to the security policy mentioned in Saadaoui et al. (2014). Table 19 also shows that the ACOFAME has produced fewer rules compared with the approach of Saadaoui et al. (2014) but at the same time conforming to the security policy.

**Table 17:** List of source addresses and their trust factors assigned based on the topology and security policy given in Saadaoui et al. (2014)

Source IP address	
172.16.0.22/30	0
10.0.0.1	0.4
10.0.0.0/16	0
10.1.0.0/16	0
192.168.0.0/24	0.4
192.168.1.0/24	0

**Table 18:** Risk values based on security policy

Destination IP address	
172.16.0.22/30	8
10.0.0.0/16	5
10.1.0.0/16	5
192.168.0.0/24	5
192.168.1.0/24	5

**Table 19:** Resolved output of ACOFAME for the ruleset of Table 3 of Saadaoui et al. (2014)

Original rule no.	Order	Action	Protocol	Port no.	SIP	DIP
R2	R1	Accept	TCP	80	10.1.0.0/16	172.16.0.22/30
R4	R2	Deny	TCP	80	10.0.0.0/15	172.16.0.22/30
R3	R3	Deny	TCP	80	192.168.0.0/23	172.16.0.22/30
R9	R4	Accept	TCP	80	192.168.0.0/24	172.16.0.0/16
R8	R5	Deny	TCP	*	10.0.0.1	172.16.0.22/30

#### Results when algorithm of Saadaoui et al. (2014) is applied to ruleset of Table 2 with security policy of Table 1

The ruleset of the study listed in Table 2 was also processed through the approach proposed in Saadaoui et al. (2014). At level 1, all shadowed and redundant rules are eliminated. Rules 7, 8, 9, 12, and 13 were shadowed by rule 4 and hence were removed. Rule 1 was redundant with respect to rule 4; hence, rule 1 was eliminated. The output after level 1 is shown in Table 20. Java programming was used in the implementation.

**Table 20:** Output after level 1 of Saadaoui et al. (2014) is applied to the ruleset of Table 2 of this study

Original order	New rule no.	Protocol	Source IP	Source port	Destination IP	Destination port	Action
2	1	TCP	172.19.55.*	*	10.12.32.21	80	Allow
3	2	TCP	192.168.5.64	*	10.12.32.23	23	Allow
4	3	*	172.19.55.121–172.19.55.124	*	10.12.32.*	*	Allow
5	4	*	10.45.48.34	*	10.12.32.*	*	Deny
6	5	*	10.*.*.*	*	10.12.32.21	80	Allow
10	6	TCP	192.168.15.253	*	10.12.32.23	23	Allow
11	7	TCP	10.44.128.112	*	10.12.32.23	23	Allow
14	8	*	172.19.64.221	*	10.12.32.*	*	Deny

The output of level-1 is now applied to level-2, the conflict rule-class anomalies resolver. Rules 4 and 5 (5 and 6 in the actual input) were correlated, and based on security policy, a new rule is added before rule 5 saying allow communication through the correlated part. The final resolved output is shown in Table 21. Comparing this output with Table 11, which is the output of ACOFAME, Table 21, which was generated by Saadaoui et al. (2014) has fewer rules than does Table 11, which was generated by ACOFAME. However, rules 1, 2, 3, 4, 6, 9, 10 in Table 21 conform to the security policy of Fig. 1, whereas rules 5, 7, and 8 do not conform to the security policy. Therefore, with the help of modified TF-based RV calculations and by applying the ACO search based on the CRS, the proposed approach is feasible and has added value to existing approaches.

**Table 21:** The final resolved ruleset of input rule of Table 2 applied to the algorithm of Saadaoui et al. (2014)

Original order	New rule no.	Protocol	Source IP	Source port	Destination IP	Destination port	Action
2	1	TCP	172.19.55.*	*	10.12.32.21	80	Allow
3	2	TCP	192.168.5.64	*	10.12.32.23	23	Allow
4	3	*	172.19.55.121–172.19.55.124	*	10.12.32.*	*	Allow
New	4	*	10.45.48.34	*	10.12.32.21	80	Allow
5	5	*	10.45.48.34	*	10.12.32.*	*	Deny
6	6	*	10.*.*.*	*	10.12.32.21	80	Allow
10	7	TCP	192.168.15.253	*	10.12.32.23	23	Allow
11	8	TCP	10.44.128.112	*	10.12.32.23	23	Allow
14	9	*	172.19.64.221	*	10.12.32.*	*	Deny

## Conclusion and future scope

In this study, an ACOFAME was proposed that can automatically detect and resolve packet-filter firewall anomalies. A relationship between the Security Policy and the resolver was established by introducing the concept of TF. The TF-based Action Constraint Generation has reduced the Availability Loss and increased the chance of the resolved ruleset conforming to the security policy. The bio-inspired Ant Colony Optimization algorithm proved successful in finding the best possible reordering of firewall rules, which can resolve more conflicts than existing methods can at a cost of increased computational time for larger rule sizes. ACOFAME also eliminated the need for a separate rule redundancy phase. The adaptive reordering technique will be helpful in reducing the significant amount of time required to mitigate anomalies when a new rule is appended. This research proposes a practically feasible and implementable solution that will be of great help to administrators and organizations that maintain a packet-filter firewall. A limitation that is worth mentioning is that the source IP address can be subject to spoofing attacks and must be addressed separately. As a future work, other bio-inspired optimization techniques can be considered and compared. This work can also be extended to distributed firewalls. Furthermore, research might be warranted to inquire about other possible Action Constraint Generation strategies such as High Authority Override or other techniques, and situational-based usage of strategies can be verified.
